# Efficacy and safety of rituximab in autoimmune and microangiopathic hemolytic anemia: a systematic review and meta-analysis

**DOI:** 10.1186/s40164-020-00163-5

**Published:** 2020-04-15

**Authors:** Shih-Hsuan Chao, Yuh-Lih Chang, Jiin-Cherng Yen, Hsien-Tzung Liao, Tsai-Hung Wu, Chia-Li Yu, Chang-Youh Tsai, Yueh-Ching Chou

**Affiliations:** 1 Institute of Pharmacology, National Yang-Ming University, Taipei, Taiwan; 2 Department of Pharmacy, Taipei Veterans General Hospital, Taipei, Taiwan; 3 Division of Allergy Immunology & Rheumatology, Taipei Veterans General Hospital, 201 Shih-Pai Rd Sec 2, Taipei, 112 Taiwan; 4 Division of Nephrology, Taipei Veterans General Hospital, Taipei, Taiwan; 5 Division of Rheumatology Immunology & Allergy, Department of Internal Medicine, National Taiwan University Hospital, Taipei, Taiwan

## Abstract

**Background:**

The efficacy and safety of rituximab (RTX) on hemolytic anemia (HA) is unknown. Therefore we retrospectively analyze the efficacy and safety of RTX in autoimmune hemolytic anemia (AIHA) and microangiopathic hemolytic anemia (MAHA) from the previous literature.

**Methods:**

Data in clinical trials and observational studies were collected from PubMed, Cochrane, Embase, and Google Scholar until Oct 15, 2018. The efficacy and safety of RTX in patients with AIHA or MAHA were assessed and overall response rates (ORRs), complete response rates (CRRs), adverse events (AEs) and relapse rates (RRs) were extracted if available. A meta-analysis was performed with a random-effects model, estimating mean proportions in all studies, and relative rates in comparative studies.

**Results:**

After quality assessment, a total of 37 investigations encompassing 1057 patients eligible for meta-analysis were included. Pooled mean proportion of ORR was 0.84 (95% confidence interval [CI] 0.80–0.88), and that of CRR was 0.61 (95% CI 0.49–0.73). Mean AE rate was 0.14 (95% CI 0.10–0.17), and mean RR was 0.21 (95% CI 0.15–0.26). Relative ORR was 1.18 (95% CI 1.02–1.36), and relative CRR was 1.17 (95% CI 0.98–1.39) fold more than the respective non-RTX counter parts. Relative AE rate was 0.77 (95% CI 0.36–1.63), and relative RR was 0.93 (95% CI 0.56–1.55) fold less than the respective non-RTX counter parts.

**Conclusion:**

RTX is more effective than the treatments without RTX for AIHA and MAHA and is well-tolerated.

## Introduction

Hemolytic anemia (HA) is an anemia due to premature destruction of erythrocytes (or red blood cells, RBCs) in the circulation before their normal demise [1]. Its diagnosis is based on decreased hemoglobin and/or haptoglobin, increased reticulocytes, indirect bilirubin, and lactate dehydrogenase, as well as typical findings demonstrated in peripheral blood smear [1, 2]. There are several causes of HA, which may result in erythrocyte destruction occurring at different locations, such as in larger vessels in the case of autoimmune hemolytic anemia (AIHA) or in smaller vessels in the case of microangiopathic hemolytic anemia (MAHA) [2].

AIHA is caused by autoantibodies against self-antigens in erythrocytes, leading to a premature RBC destruction, which could be diagnosed by a positive direct antiglobulin test (DAT) [3]. AIHA can be subdivided into warm, mixed, or cold-reactive subtypes, according to the optimal autoantibody-RBC reactivity temperatures [4–6]. Treatment options depend on the types of AIHA, with corticosteroids or supportive care as the mainstay of the treatment [7].

MAHA is a descriptive term for non-immune hemolysis resulting from intravascular RBC fragmentation [8]. Its main difference from AIHA is that patients with MAHA get negative in DAT [8]. The prominent causes of MAHA are thrombotic thrombocytopenic purpura (TTP) and hemolytic uremic syndrome (HUS) [8]. The disease can be hereditary or acquired by inhibiting autoantibodies [9]. TTP is mainly caused by a severely reduced activity of the von Willebrand factor-cleaving protease, ADAMTS13. Its standard treatment is plasma exchange (PEX) in conjunction with corticosteroids [10]. Typical infection-associated HUS is triggered by Shiga toxin producing *Escherichia coli*, and primary HUS is caused by complement dysregulation. The standard treatment is eculizumab for HUS [11].

Rituximab (RTX) is a B cell depleting monoclonal antibody which binds to cluster of differentiation (CD) 20 expressed on the surface of B cell that produces anti-RBC antibodies. It has been used as a therapeutic biologic agent in B cell lymphoma and leukemia [12]. A systematic review has discussed about safety and efficacy of RTX in immune-mediated disorders [13]. A meta-analysis has shown high short-term benefit/risk ratio of RTX in AIHA [14]. A randomized controlled trial (RCT) has revealed that RTX combined with prednisone may confer a better benefit/risk ratio than prednisone alone for treating adults with newly-diagnosed warm-type AIHA (wAIHA) [15]. A phase 2 study showed a high remission and survival rate for TTP patients treated with PEX and RTX [16]. Overall, RTX may be effective in AIHA or, presumably in MAHA. However, the evidence of favorability for this treatment modality is not yet clear. To further investigate the efficacy and safety of RTX in AIHA and MAHA, we conducted this systematic review and meta-analysis.

## Methods

### Data sources and search strategy

All stages of the present investigation followed the Meta-analysis Of Observational Studies in Epidemiology (MOOSE) and Preferred Reporting Items for Systematic reviews and Meta-Analyses (PRISMA) guidelines [17, 18]. An exhaustive literature search was carried out on PubMed, Cochrane Library, Embase, and Google Scholar from the inception to Oct 15, 2018. The search criteria included AIHA, MAHA, thrombotic microangiopathy (TMA), TTP or HUS and RTX in Medical Subject Heading (MeSH), limiting to human studies.

### Study selection

We included clinical trials or observational studies published in English, which assessed the efficacy and safety of RTX in AIHA or MAHA (TTP/HUS). Duplicated investigations, studies in other research scopes or without desired outcomes, enrollment with insufficient sample size of less than 10 patients, and those without full article publication were excluded.

### Data extraction

The extracted data included sample size, population, age, gender, dosage and framework of RTX treatment, the first author’s name, year of publication, study design, time of first evaluation, mean follow-up time and definition of treatment efficacy. The number of patients with overall response (OR) and/or complete response (CR) was extracted from each study. When available, the eventual relapse, side effects or toxicities were also collected.

### Quality assessment

Quality was assessed using the revised and validated version of Methodological Index for NOn-Randomized Studies (MINORS) [19]. Non-comparative studies were scored on clearly stated aim, inclusion of consecutive patients, prospective collection of data, appropriate endpoints, unbiased assessment of the study endpoint, appropriate follow-up time period, loss to follow-up of less than 5%, and prospective calculation of the study size. Additional criteria for comparative studies were an adequate control group, contemporary control groups, baseline equivalence of groups, and adequate statistical analyses. Studies received 0 to 2 points for each of these components. The total score ranged from 0 to 24 points for comparative studies and 0 to 16 points for non-comparative studies. Scores no less than 18 in comparative studies and no less than 12 in non-comparative studies were considered to be with high quality and were included in quantitative analysis.

### Data synthesis and analysis

Efficacy was assessed by overall response rate (ORR) and complete response rate (CRR). Weighted mean proportions of response rate were calculated over all studies that included in the quantitative analysis, and relative ORR and relative CRR were calculated over comparative studies. As for the safety, adverse events (AEs) were extracted from all included studies. The  weighted mean relapse rate (RR) in all studies and relative RR in comparative studies were calculated. We used a random-effects model to control the between-study variance and to produce an overall summary as described by Dersimonian and Laird [20]. R Software (package metafor, version 2.0-0) was used for all analyses.

We assessed statistical heterogeneity by using I^2^ statistic and Cochran’s Q test. An I^2^ statistic with values greater than 50% or Q test with *p*-value less than 0.1 indicates substantial heterogeneity. To explain heterogeneity, the effects of covariates on response rate were investigated using mixed-effects meta-regression. The risk of publication bias was determined by the method of funnel plot and Egger’s regression test, which plots the natural log of effect sizes versus their standard error, and linear regression of funnel plot test asymmetry.

## Results

### Study selection

**Fig. 1:**
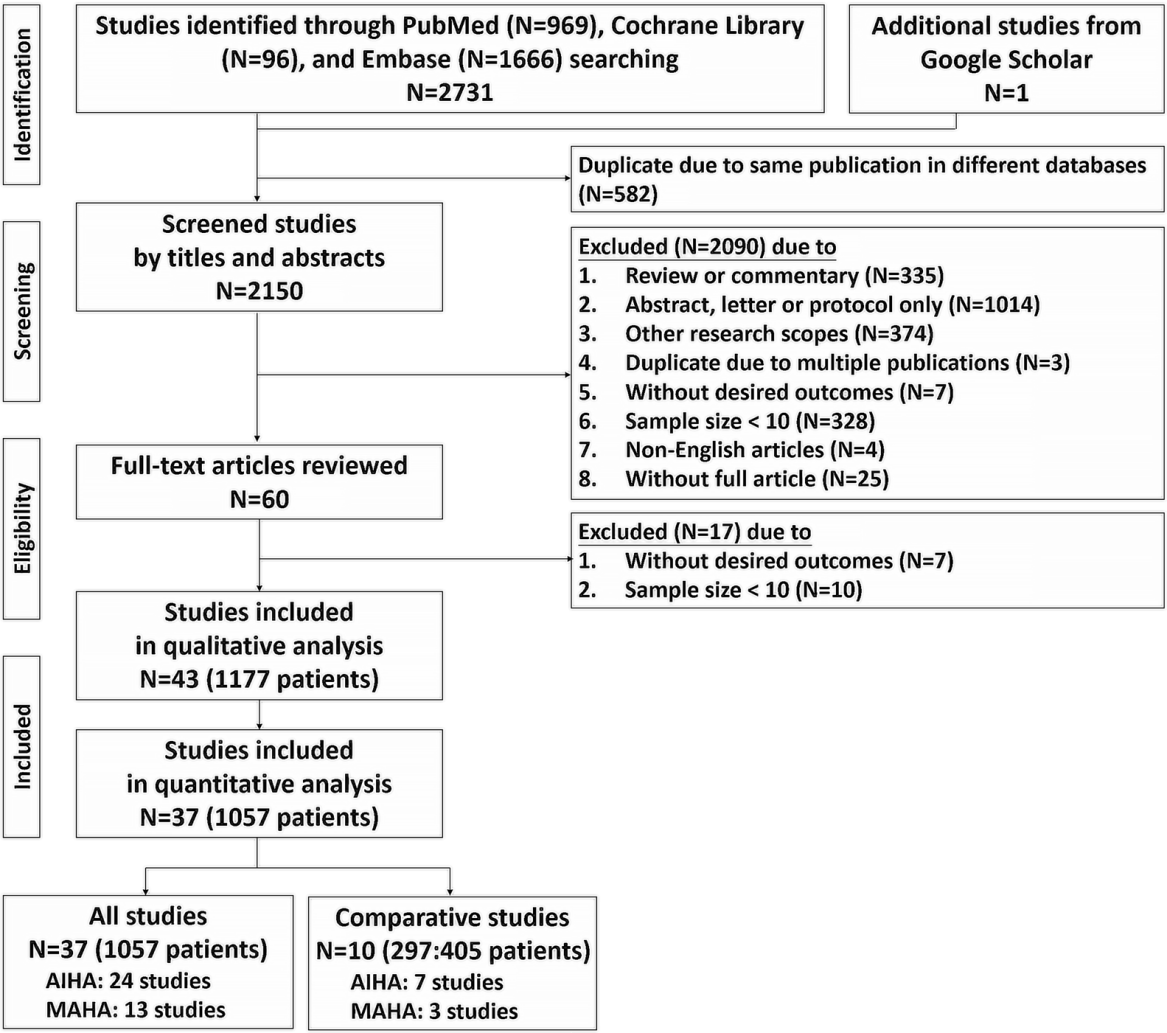
Flow chart of the systematic literature review with meta-analysis

Figure 1 summarized the flow chart of the systematic literature review with meta-analysis. A total of 2732 articles were initially identified through electronic searching. After duplication of the same publications in different databases were removed, the titles and abstracts of the remaining studies were reviewed, and 60 articles with potentially relevant studies were further identified in full-text. Finally, 43 published studies were determined to be eligible and were included in the qualitative analysis. Among 43 studies included in the qualitative analysis, 37 studies reached the cut point of high quality and were included in the quantitative analysis [15, 16, 21–55].

### Description of the studies

Characteristics of the included studies are shown in Table 1. Among 37 studies included in the quantitative analysis, 24 studies dealt with AIHA, while 13 included MAHA patients. Most of them were observational studies, and 10 of them were comparative studies, encompassing 7 AIHA studies and 3 MAHA studies. Sample size varied from 10 to 86 patients, the mean age was 50.6 years old, and the mean proportion of male was 41.0%. Patients mostly received four weekly infusions of RTX at 375 mg/m^2^. Time of the first evaluation was 0.2 month to 1 year, and the follow-up time ranged from 6 to 83 months.

**Table 1:** Characteristics of studies included in quantitative analysis

Disease	AIHA	MAHA
All studies 37 studies 1057 patients	24 studies 746 patients	13 studies 311 patients
	wAIHA 8 studies 182 patients	TTP 12 studies 297 patients
	CAD 5 studies 173 patients	HUS 1 study 14 patients
Comparative studies 10 studies 297:405 patients	7 studies 232:303 patients	3 studies 65:102 patients

Publication year: 2004–2018; sample size: 10–86 (mean age: 50.6, mean male proportion: 41.0%); rituximab regimen: 28 with 375 mg/m^2^ 4 weekly, 8 with other regimen (e.g. 100 mg 4 weekly); study design: 13 clinical trials, 24 observational studies; 1st evaluation time: 0.2 months–1 year; follow-up time: 6–83 months

### Efficacy in all studies

**Fig. 2:**
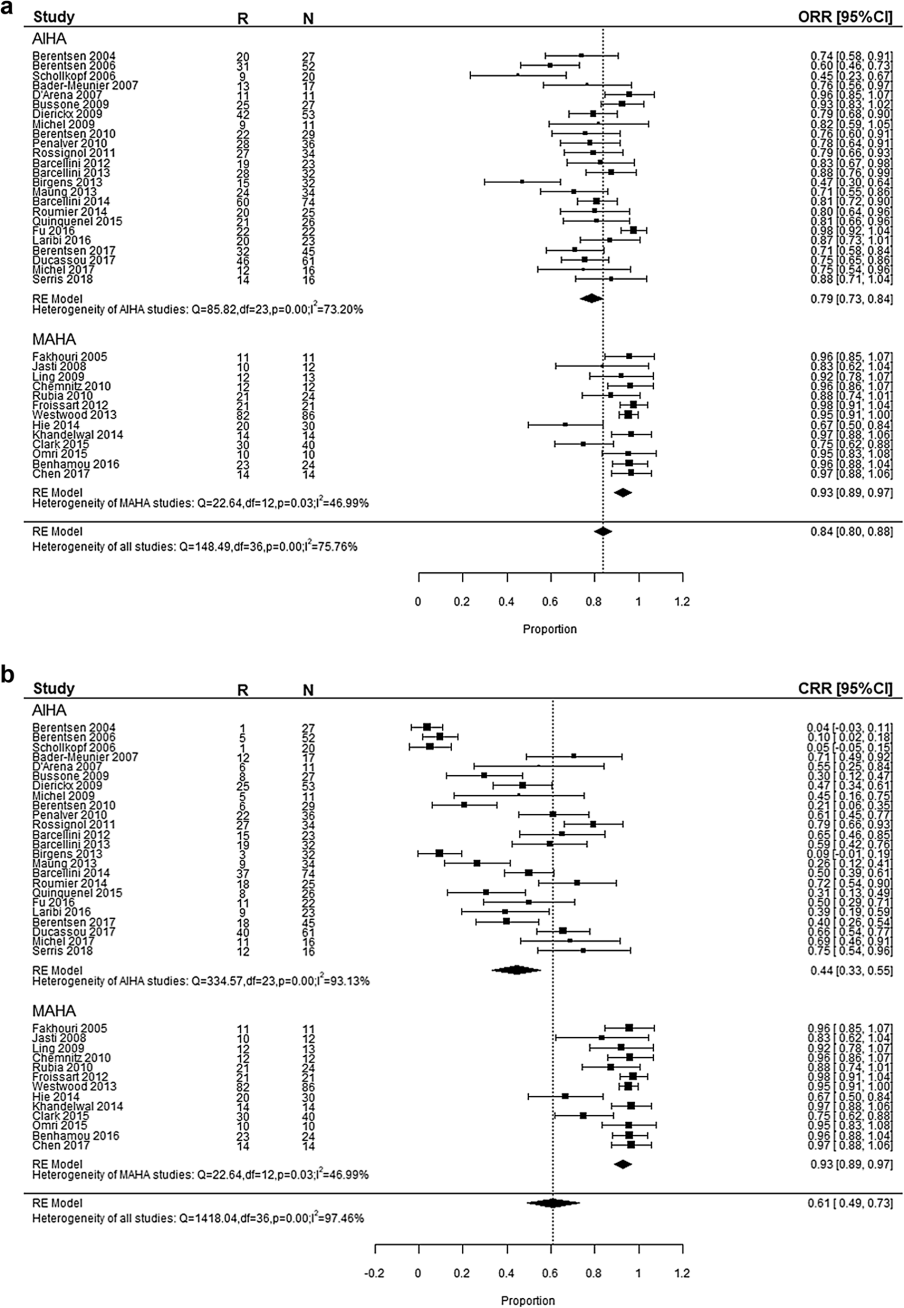

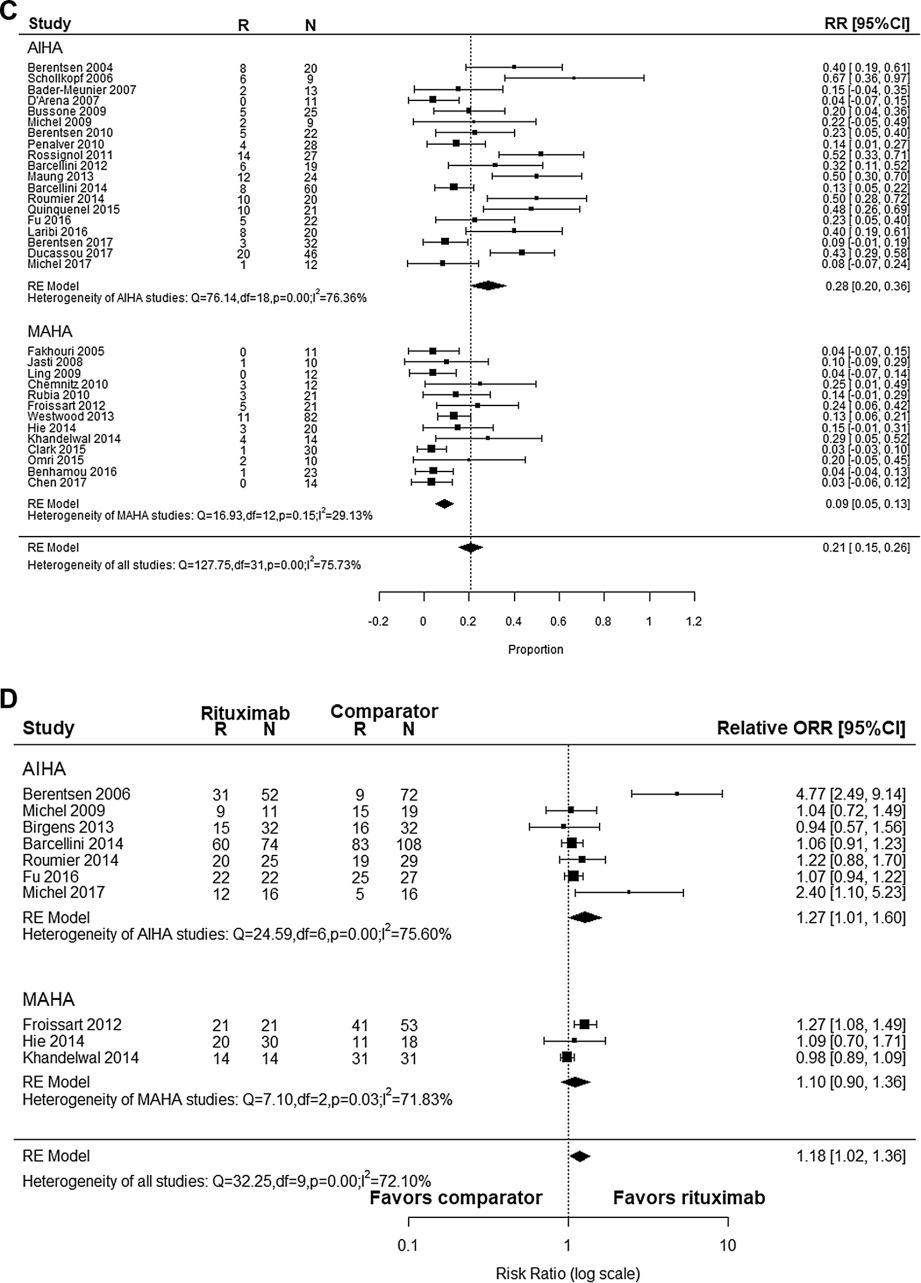

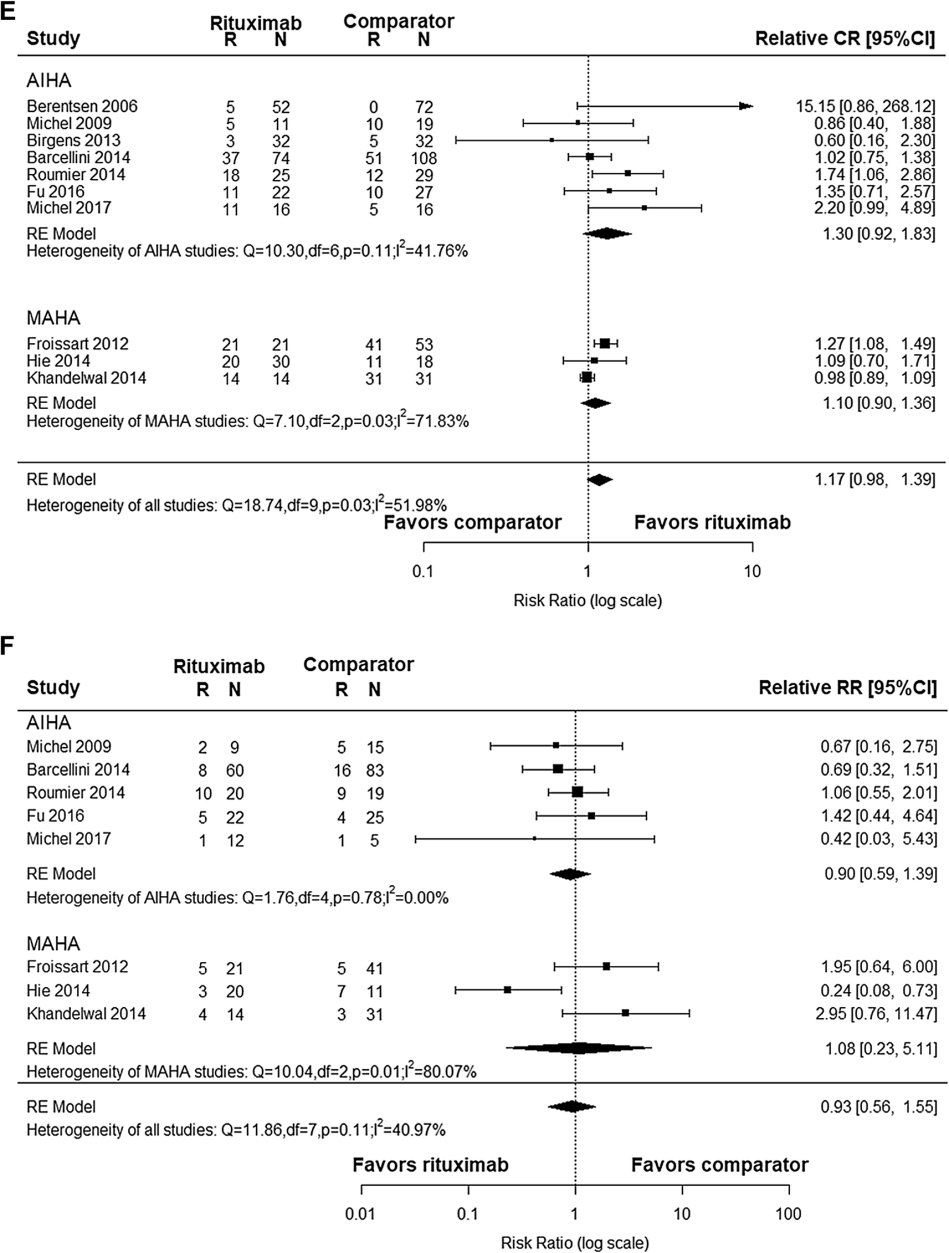
Forest plot. **a** Overall response rate of rituximab in AIHA and MAHA. **b** Complete response rate of rituximab in AIHA and MAHA. **c** Relapse rate of rituximab in AIHA and MAHA. **d** Relative overall response rate of rituximab in AIHA and MAHA. **e** Relative complete response rate of rituximab in AIHA and MAHA. **f** Relative relapse rate of rituximab in AIHA and MAHA

**Fig. 3:**
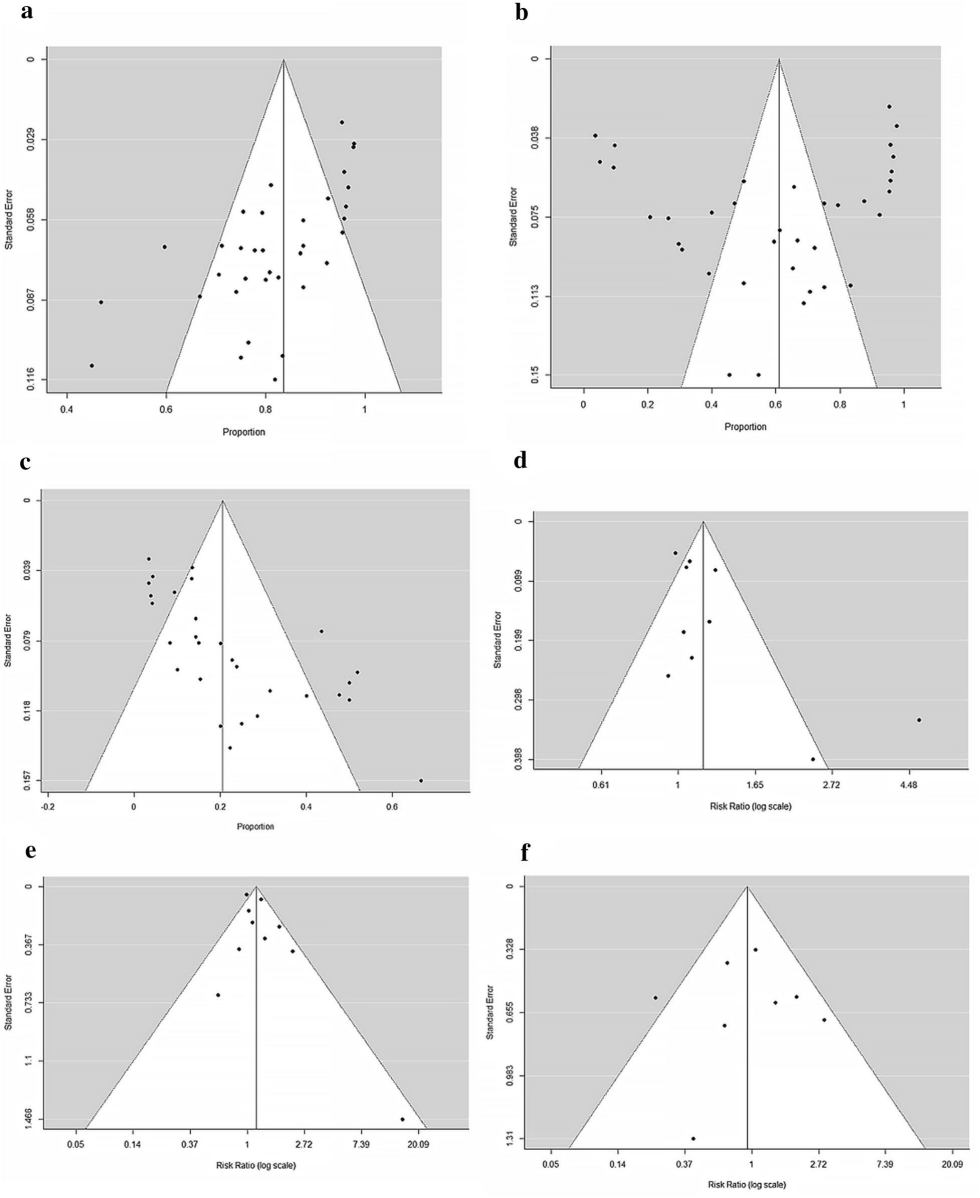
Funnel plot. **a**. Publication bias of overall response rate of rituximab in AIHA and MAHA. **b** Publication bias of complete response rate of rituximab in AIHA and MAHA. **c** Relapse rate of rituximab in AIHA and MAHA. **d** Publication bias of relative overall response rate of rituximab in AIHA and MAHA. **e** Publication bias of relative complete response rate of rituximab in AIHA and MAHA. **f** Publication bias of relative relapse rate of rituximab in AIHA and MAHA

After pooling the effect of 37 studies, we got the weighted mean proportion of response rate. The ORR was 84% (95% CI 0.80–0.88) with high heterogeneity (I^2^ = 75.76%, *p* < 0.01), as shown in Fig. 2a. Funnel plot of ORR was asymmetrical as shown in Fig. 3a, which indicated possible publication bias (Egger’s regression test, *p* < 0.0001). The CRR was 61% (95% CI 0.49–0.73) with significant heterogeneity (I^2^ = 97.46%, *p *< 0.01), as shown in Fig. 2b. Funnel plot of CRR was symmetrical, indicating no publication bias (Fig. 3b, Egger’s regression test, *p* = 0.631).

Subgroup analyses were done because of the high heterogeneity, which were shown in Table 2 in detail. For AIHA, ORR was 79% (95% CI 0.73–0.84) and CRR was 44% (95% CI 0.33–0.55). Eight studies provided data for wAIHA with ORR of 79% (95% CI 0.69–0.90) and CRR was 49% (95% CI 0.22–0.77). For cold agglutinin disease (CAD), ORR was 66% (95% CI 0.57–0.76) and CRR was 14% (95% CI 0.03–0.25). As for MAHA, the response rate was 93% (95% CI 0.89–0.97). Further dividing MAHA into TTP and HUS, the response rate was 92% (95% CI 0.88–0.96) in TTP and 97% (95% CI 0.88–1.06) in HUS respectively. There was no significant difference wherever these data came from with regards to the study design or dosage regimen. All treatment modalities in MAHA studies were in combination with PEX or plasma infusion. Therefore, we couldn’t analyze the impact of different treatment modalities in MAHA studies. But the response rate of RTX alone were similar to combination therapy in AIHA (RTX alone: 82% in OR, 44% in CR; treatments in combination: 74% in OR, 45% in CR).

**Table 2:** Subgroup analysis

Subgroup category	OR rate			CR rate			RR		
	Study, n	ORR (95% CI)	I^2^ (%)	Study, n	CRR (95% CI)	I^2^ (%)	Study, n	RR (95% CI)	I^2^ (%)
Overall	37	0.84 (0.80, 0.88)	75.76	37	0.61 (0.49, 0.73)	97.46	32	0.21 (0.15, 0.26)	75.73
Study design									
Clinical trial	13	0.80 (0.72, 0.89)	83.81	13	0.56 (0.32, 0.80)	98.54	11	0.15 (0.07, 0.22)	73.10
Observational study	24	0.85 (0.80, 0.89)	69.89	24	0.64 (0.51, 0.76)	95.79	21	0.23 (0.17, 0.30)	72.29
Diagnosis									
AIHA	24	0.79 (0.73, 0.84)	73.20	24	0.44 (0.33, 0.55)	93.13	19	0.28 (0.20, 0.36)	76.36
wAIHA	8	0.79 (0.69, 0.90)	75.89	8	0.49 (0.22, 0.77)	96.27	7	0.31 (0.14, 0.47)	82.22
CAD	5	0.66 (0.57, 0.76)	45.99	5	0.14 (0.03, 0.25)	82.57	4	0.31 (0.10, 0.53)	81.81
MAHA	13	0.93 (0.89, 0.97)	46.99	13	0.93 (0.89, 0.97)	46.99	13	0.09 (0.05, 0.13)	29.13
TTP	12	0.92 (0.88, 0.96)	50.74	12	0.92 (0.88, 0.96)	50.74	12	0.08 (0.04, 0.12)	21.38
HUS	1	0.97 (0.88, 1.06)		1	0.97 (0.88, 1.06)		1	0.29 (0.05, 0.52)	
Dosage regimen									
375 mg/m^2^ 4 weekly	28	0.83 (0.78, 0.88)	78.64	28	0.62 (0.48, 0.77)	98.03	24	0.20 (0.14, 0.26)	77.50
Other regimen	8	0.84 (0.76, 0.91)	67.49	8	0.56 (0.45, 0.67)	70.80	7	0.24 (0.13, 0.32)	72.87
Treatment modalities of AIHA									
RTX alone	14	0.82 (0.76, 0.88)	72.74	14	0.44 (0.29, 0.59)	93.22	12	0.30 (0.20, 0.40)	78.27
In combination	10	0.74 (0.66, 0.81)	59.22	10	0.45 (0.27, 0.63)	93.70	7	0.26 (0.12, 0.39)	76.19

**Fig. 4:**
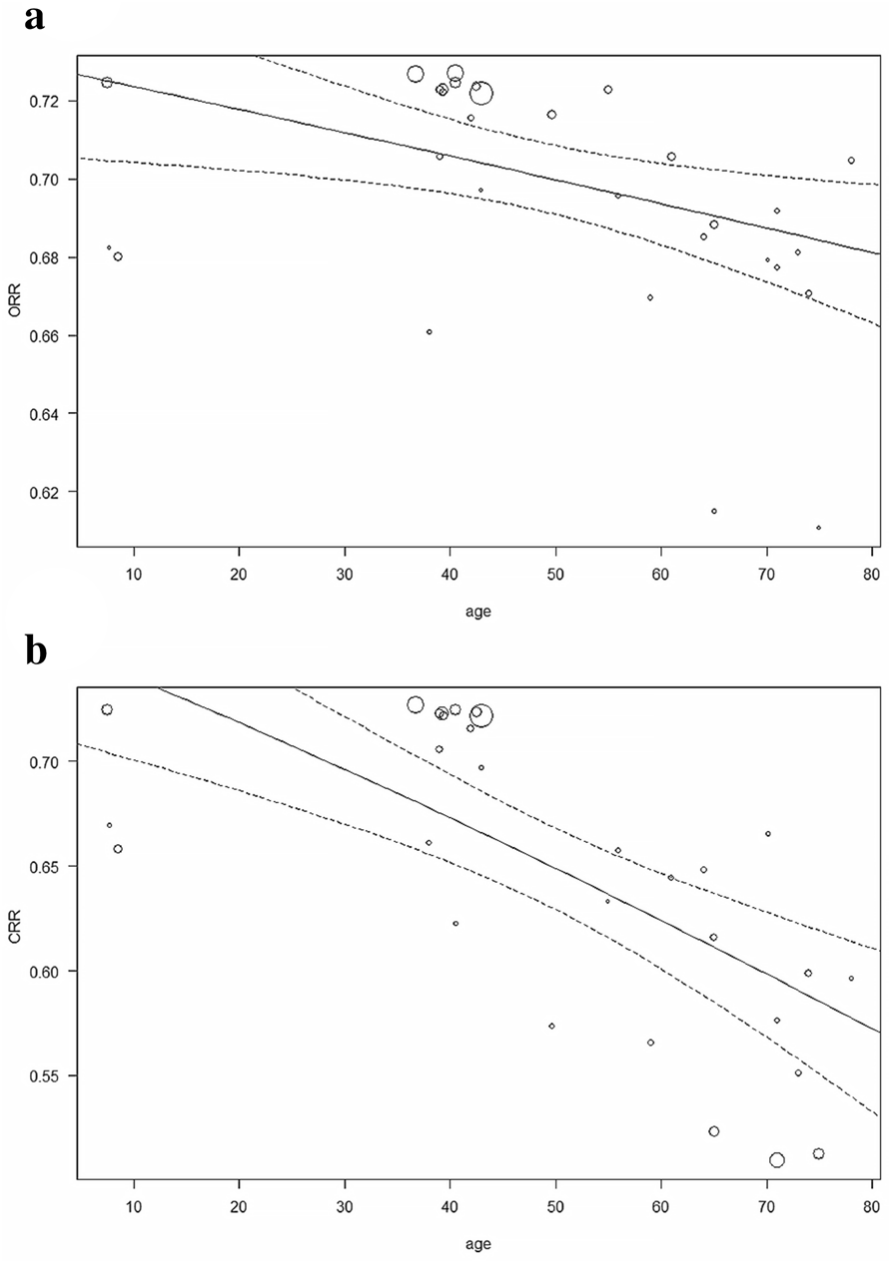
Bubble plot. **a** Relation of age and overall response rate. **b** Relation of age and complete response rat

We further carried out meta-regression to find out another factors that may influence the response rate (Table 3). Among all the factors we tested, ORR and CRR were only significantly associated with age (ORR: *r*^2^ = 23.29, *p* = 0.0103; CRR: *r*^2^ = 45.37, *p* < 0.0001) as shown in Fig. 4a, b, respectively.

**Table 3:** Meta-regression

	OR rate			CR rate			RR		
	Study (n)	R^2^ (%)	*p*-value	Study (n)	R^2^ (%)	*P*-value	Study (n)	R^2^ (%)	*p*-value
Sample size	37	5.11	0.1079	37	1.29	0.2262	32	0.00	0.7044
Year of publication	37	0.00	0.3522	37	5.83	0.0905	32	0.00	0.9247
Age	30	23.29	0.0103	30	45.37	< 0.0001	27	0.00	0.3689
Proportion of male	30	0.00	0.6095	30	0.00	0.5294	27	0.00	0.4814
Evaluation time	29	3.70	0.2370	29	0.00	0.4967	27	0.00	0.6003
Follow-up time	37	8.59	0.1167	37	0.00	0.9396	32	0.00	0.6639
Quality score	37	0.00	0.1886	37	0.10	0.3369	32	0.42	0.3695

Among 872 patients included, there were 163 relapse events, with an overall RR of 21% (95% CI 0.15–0.26) (Fig. 2c). Because funnel plot for RR was asymmetrical (Fig. 3c), it is possible that there is a publication bias (Egger’s regression test, *p* < 0.0001). In a further subgroup analysis (Table 2), RR was 28% (95% CI 0.20–0.36) in AIHA and 9% (95% CI 0.05–0.13) in MAHA respectively. However, meta-regression failed to demonstrate any other additional confounding factor (Table 3).

### Safety in all studies

Briefly, 32 studies reported 138 AEs in 851 patients. The overall AE rate was 13% (95% CI 0.10–0.17). Among them, 33 events (23.9%) were infusion-related reactions, such as fever, chills, skin rash, and allergic reactions, 34 events (24.6%) were infections, and 40 events (29.0%) were hematologic abnormalities, mostly neutropenia. Besides, there were 3 mortalities (0.3%), including one early death from uncontrolled hemolysis [38]. The 2nd fatal patient died because of delayed treatment [55]. The 3rd fatal patient died shortly after RTX therapy, which was also claimed to be partially contributing to the death [42]. Importantly, there is a critical issue about hepatitis B virus reactivation (HBVr) regarding AE of RTX. There was no records about it in the present meta-analysis. The previous epidemiological study provided prevalence estimates for a mean global HBsAg prevalence of 4.9% [56]. In Taiwan, the mean HBsAg prevalence goes to 13.7% [56]. So, rationale for the absence of HBVr as AE in the present investigation may be that the included studies were all from Western countries. It is conceivable that quite different conclusion might be reached if the studies are conducted using Taiwanese data, which will be interesting.

### Efficacy in comparative studies

After pooling the effect of 10 comparative studies, we got the relative response rate. As shown in Fig. 2d, relative OR was 1.18 (95% CI 1.02–1.36) with high heterogeneity (I^2^ = 72.10%, *p *< 0.01). Funnel plot for relative OR was asymmetrical as shown in Fig. 3d, indicating possible publication bias (Egger’s regression test, *p* = 0.0056). On the other hand, relative CR was 1.17 (95% CI 0.98–1.39) with heterogeneity (I^2^ = 51.98%, *p *= 0.03, Fig. 2e) and with a symmetrical funnel plot indicating the absence of publication bias (Egger’s regression test, *p* = 0.1736, Fig. 3e).

Sensitivity analysis was carried out via leave-one-out approach in both relative ORR and relative CRR. In the former, leaving “Berentsen et al. 2006” out resulted in the reduction in the overall heterogeneity significantly, coming to an ORR of 1.09 (95% CI 1.00–1.19). On the other hand, leaving “Khandelwal et al. 2014” out led to a reduction in overall CRR heterogeneity significantly, coming to 1.24 (95% CI 1.03–1.49).

Among 10 comparative studies, 8 studies reported RR. The overall relative RR was 0.93 (95% CI 0.56–1.55, Fig. 2f) with a symmetrical funnel plot (Egger’s regression test, *p* = 0.8543, Fig. 3f), indicating that there were no publication bias. Regarding the sensitivity analysis (Table 4), leaving “Hie et al. 2014” out resulted in reduction in the overall heterogeneity significantly, coming to 1.09 (95% CI 0.74, 1.60).

**Table 4:** Sensitivity analysis

No.	Study that leave	OR rate			CR rate			RR		
		Relative rate (95% CI)	I^2^ (%)	*p*-value	Relative rate (95% CI)	I^2^ (%)	*p*-value	Relative rate (95% CI)	I^2^ (%)	*p*-value
A4	Berentsen (2006)	1.09 (1.00, 1.19)	34.44	0.14	1.15 (0.98, 1.35)	48.37	0.05			
A12	Michel (2009)	1.20 (1.02, 1.40)	75.15	0.00	1.19 (0.99, 1.43)	56.56	0.02	0.96 (0.55, 1.71)	48.42	0.07
A18	Birgens (2013)	1.20 (1.03, 1.39)	74.94	0.00	1.18 (0.99, 1.41)	55.56	0.02			
A20	Barcellini (2014)	1.22 (1.02, 1.45)	75.07	0.00	1.21 (0.98, 1.48)	56.95	0.02	0.99 (0.54, 1.83)	46.25	0.08
A21	Roumier (2014)	1.18 (1.01, 1.37)	74.80	0.00	1.12 (0.95, 1.33)	47.14	0.06	0.90 (0.47, 1.73)	48.42	0.07
A23	Fu (2016)	1.22 (1.02, 1.46)	75.17	0.00	1.16 (0.97, 1.40)	56.23	0.02	0.88 (0.49, 1.56)	46.94	0.08
A27	Michel (2017)	1.15 (1.00, 1.32)	71.68	0.00	1.13 (0.96, 1.34)	48.94	0.05	0.96 (0.56, 1.65)	47.72	0.07
M7	Froissart (2012)	1.16 (0.99, 1.37)	71.24	0.00	1.15 (0.93, 1.41)	40.60	0.10	0.83 (0.49, 1.56)	39.83	0.13
M9	Hie (2014)	1.19 (1.02, 1.38)	75.20	0.00	1.18 (0.98, 1.44)	57.31	0.02	1.09 (0.74, 1.60)	0.00	0.47
M10	Khandelwal (2014)	1.23 (1.04, 1.46)	70.04	0.00	1.24 (1.03, 1.49)	25.52	0.22	0.83 (0.50, 1.35)	32.18	0.18

### Safety in comparative studies

Among 10 comparative studies, 6 studies documented AEs in both groups. In RTX group, there were 34 AEs in 130 patients, and there were 55 AEs in 188 patients in comparative group. The relative AE rate was 0.74 (95% CI 0.33–1.63) for RTX.

## Discussion

The results in the present “efficacy in AIHA study” were consistent with those reported previously [14]. However, our “efficacy in wAIHA” was higher than those reported in cold agglutinin disease (CAD). This implies that warm type AIHAs are more likely to be resulted from B cell hyperactivity. Moreover, the present study on “the efficacy of RTX in MAHA” is the first one to our knowledge. We have found that the response rate of RTX was higher in MAHA. Furthermore, these effects were the same in patients either with TTP or HUS. The reason for this is unclear because apparently MAHA is not developing on the basis of deranged B cell regulation. However, HUS or TTP has been reported to be originated from autoantibodies such as ant-dsDNA, anticardiolipin antibodies, &/or other autoantibodies against ADAMS13 [57, 58].

In subgroup analysis, regimens with different dosage of RTX showed a similar response rate. Most studies used the regimen of 375 mg/m^2^ 4-weekly, which might contribute to the effects. These results are interesting because monoclonal antibody drugs are quite different from traditional small molecule drugs in that they are acting on the cells via the receptors or ligands. Since the cells usually have their own threshold or affinity limitation, the traditional dose–response relationship cannot be applied to this scenario. Moreover, the initial effect of the biologics on the cell can even be amplified or reduced via cell–cell contact or crosstalk. Further investigations may be necessary to determine the optimal dosage for RTX in AIHA and MAHA. When it comes to different treatment modalities, response rate of RTX alone were similar to combination therapy in AIHA, i.e., RTX alone can be reasonably considered in patients with AIHA. Furthermore, regarding the effect of age on the response rate of RTX in HA patients, we found that the response rate was significantly reduced along with aging of the recipients. It may imply that senility is aligned with degeneration of the immunity such that the immune therapy on B cells is also turning to be ineffective. However, it is also possible that younger patients present with a more benign form of the disease. As for RR, our result in AIHA (28%) were consistent with those from previous meta-analysis (27%) [14]. Patients with MAHA exhibited lower RR (9%) in the present study. Although there were some patients with relapse in this group, most of them recovered after repeating with RTX.

RTX seems safe and well-tolerated in AIHA and MAHA. This was consistent with previous meta-analysis [14]. Few AEs have been reported (13%) in all of our included studies and even when they were present, the majority of them were otherwise mild. However, there are some common AEs in the in-label use of RTX, which were more than 25% in incidence, such as infusion reactions. Overall, RTX showed lower AE rate in AIHA and MAHA than in other diseases.

On the other hand, there is a critical issue about hepatitis B virus reactivation (HBVr) regarding AE of RTX. Indeed, there was no records for this in the present meta-analysis. In the epidemiological studies, the average global prevalence of HBV infection is 4.8% whereas the figure is as high as 23.7% in Taiwan, which is among the highest in the Asia–Pacific region [55]. The absence of HBV related AE in the present meta-analysis is conceivable because the included studies were all carried out in Western countries. If the studies done in Asia–Pacific region including Taiwan were included, the results are expected to be quite different, necessitating further relevant investigations.

The present investigation has provided overwhelming evidence for the first time that RTX is effective for AIHA and MAHA, compared to the conventional treatment modalities that did not include RTX. A previous meta-analysis only estimated the pooled mean response rate of RTX in AIHA] [14]. In sensitivity analysis of ORR, getting rid of Berentsen’s data in 2006 could reduce the overall heterogeneity significantly [22]. This is because they used a quite different comparators to assess the effect of RTX. Similarly, in sensitivity analysis of CRR, getting rid of Khandelwal’s data in 2014 resulted in a significant reduction of the overall heterogeneity [52]. The cause of it may be that their data only included HUS. Nevertheless, the response rate still favored RTX in both MAHA and AIHA.

As for the RR, there were no differences between treatments with and without RTX. In sensitivity analysis, getting rid of Hie’s data in 2014 resulted in reduction in the overall heterogeneity significantly. Their investigation was the only study significantly favoring rituximab with low RR. The follow-up time of their data was the longest (5 years), which has indicated that the long-term effect of RTX is better than that of standard cares. However, the RRs were still not different between two groups after leaving “Hie et al. 2014” out.

In the analysis of comparative studies, the AE rates were not different between treatments with and without RTX. Taken together, the safety of RTX seemed similar to that of the conventional treatment modalities.

In spite of the significant findings demonstrated in the present meta-analysis, there were still several limitations in it. Firstly, although we have searched extensively in the 4 databases for all the relevant studies, we could only found 2 randomized controlled trials. Although this has been inevitable because AIHA and MAHA are rare diseases in real life, it may cause a lower level of evidence. Secondly, our overall results showed high heterogeneity. To figure out the reasons for this, we carried out meta-regression and found that age might have been the factor that influences the response rate. The various comparators in comparative studies may have also contributed. Lastly, although our analytic process followed the MOOSE and PRISMA guidelines, publication bias existed in the estimation of ORR and RR. There are several reasons to result in the publication bias. For example, inclusion criteria in a meta-analysis may be biased if the selection is restricted to published trials or to trials published in English language journals [59]. On the other hand, most research with published bias lack studies with poor results, since studies with poor results are most likely not to be published. It may cause overestimation of the pooled effects. However, the studies that are lacking in our research are studies with better results, i.e., the lack of those studies in our research may only cause further underestimation of the effect. Hence, our results should have been convincing.

## Conclusion

RTX shows a high overall effect (84%) in AIHA and MAHA, and an even higher effect (93%) in MAHA subgroup. It also shows a better effect in both AIHA and MAHA in comparison with other treatment modalities. Moreover, the RR of the diseases after RTX treatment might have been low. Finally, RTX is safe and well-tolerated for the treatment of HA. Few AEs have been reported with most of them being mild, and the AE rate was similar to those reported in other treatment modalities.

## Data Availability

All data generated or analyzed during this study are included in the manuscript.

## Abbreviations

ADAMTS13: A disintegrin and metalloproteinase with a thrombospondin type 1 motif, member 13
AE: Adverse event
AIHA: Autoimmune hemolytic anemia
CAD: Cold agglutinin disease
CD: Cluster of differentiation
CRR: Complete response rate
DAT: Direct antiglobulin test
HA: Hemolytic anemia
HUS: Hemolytic-uremic syndrome
MAHA: Microangiopathic hemolytic anemia
MeSH: Medical Subject Headings
MINORS: Methodological Index for NOn-Randomized Studies
MOOSE: Meta-analysis Of Observational Studies in Epidemiology
ORR: Overall response rate
PRISMA: Preferred Reporting Items for Systematic reviews and Meta-Analyses
RBC: Red blood cell
RR: Relapse rate
RTX: Rituximab
TMA: Thrombotic microangiopathy
TTP: Thrombotic thrombocytopenic purpura
vWF: von Willebrand factor
wAIHA: Warm-reactive autoimmune hemolytic anemia

## References

[1] Dhaliwal G, Cornett PA, Tierney LM (2004). Hemolytic anemia. Am Fam Physician.

[2] Barcellini W, Fattizzo B (2015). Clinical applications of hemolytic markers in the differential diagnosis and management of hemolytic anemia. Dis Mark.

[3] Barcellini W (2015). Immune hemolysis: diagnosis and treatment recommendations. Semin Hematol.

[4] Bass GF, Tuscano ET, Tuscano JM (2014). Diagnosis and classification of autoimmune hemolytic anemia. Autoimmun Rev.

[5] Michel M (2011). Classification and therapeutic approaches in autoimmune hemolytic anemia: an update. Expert Rev Hematol.

[6] Berentsen S, Sundic T (2015). Red blood cell destruction in autoimmune hemolytic anemia: role of complement and potential new targets for therapy. Biomed Res Int.

[7] Hill QA, Stamps R, Massey E (2017). The diagnosis and management of primary autoimmune haemolytic anaemia. Br J Haematol.

[8] Shenkman B, Einav Y (2014). Thrombotic thrombocytopenic purpura and other thrombotic microangiopathic hemolytic anemias: diagnosis and classification. Autoimmun Rev.

[9] Tsai HM, Lian EC (1998). Antibodies to von Willebrand factor-cleaving protease in acute thrombotic thrombocytopenic purpura. NEJM.

[10] Scully M, Hunt BJ, Benjamin S (2012). Guidelines on the diagnosis and management of thrombotic thrombocytopenic purpura and other thrombotic microangiopathies. Br J Haematol.

[11] Taylor CM, Machin S, Wigmore SJ (2010). Clinical practice guidelines for the management of atypical haemolytic uraemic syndrome in the United Kingdom. Br J Haematol.

[12] Liu D, Zhao J. Frontline therapies for untreated chronic lymphoid leukemia. Exp Hematol Oncol 2019;8:15. 10.1186/s40164-019-0139-8. 10.1186/s40164-019-0139-8 PMC6698011 31428514

[13] Kaegi C, Wuest B, Schreiner J (2019). Systematic review of safety and efficacy of rituximab in treating immune-mediated disorders. Front Immunol..

[14] Reynaud Q, Durieu I, Dutertre M (2015). Efficacy and safety of rituximab in auto-immune hemolytic anemia: a meta-analysis of 21 studies. Autoimmun Rev.

[15] Michel M, Terriou L, Roudot-Thoraval F (2017). A randomized and double-blind controlled trial evaluating the safety and efficacy of rituximab for warm auto-immune hemolytic anemia in adults (the RAIHA study). Am J Hematol.

[16] Clark WF, Rock G, Barth D (2015). A phase-II sequential case-series study of all patients presenting to four plasma exchange centres with presumed relapsed/refractory thrombotic thrombocytopenic purpura treated with rituximab. Br J Haematol.

[17] Stroup DF, Berlin JA, Morton SC (2000). Meta-analysis of observational studies in epidemiology: a proposal for reporting. Meta-analysis Of Observational Studies in Epidemiology (MOOSE) group. JAMA..

[18] Moher D, Liberati A, Tetzlaff J (2009). Preferred reporting items for systematic reviews and meta-analyses: The PRISMA statement. J Clin Epidemiol.

[19] Slim K, Nini E, Forestier D (2003). Methodological index for non-randomized studies (minors): development and validation of a new instrument. ANZ J Surg.

[20] DerSimonian R, Laird N (1986). Meta-analysis in clinical trials. Control Clin Trials.

[21] Berentsen S, Ulvestad E, Gjertsen BT (2004). Rituximab for primary chronic cold agglutinin disease: a prospective study of 37 courses of therapy in 27 patients. Blood.

[22] Berentsen S, Ulvestad E, Langholm R (2006). Primary chronic cold agglutinin disease: a population based clinical study of 86 patients. Haematologia.

[23] Schöllkopf C, Kjeldsen L, Bjerrum OW (2006). Rituximab in chronic cold agglutinin disease: a prospective study of 20 patients. Leuk Lymphoma.

[24] Bader-Meunier B, Aladjidi N, Bellmann F (2007). Rituximab therapy for childhood Evans syndrome. Haematologia.

[25] D’Arena G, Califano C, Annunziata M (2007). Rituximab for warm-type idiopathic autoimmune hemolytic anemia: a retrospective study of 11 adult patients. Eur J Haematol.

[26] Bussone G, Ribeiro E, Dechartres A (2009). Efficacy and safety of rituximab in adults’ warm antibody autoimmune haemolytic anemia: retrospective analysis of 27 cases. Am J Hematol.

[27] Dierickx D, Verhoef G, van Hoof A (2009). Rituximab in auto-immune haemolytic anaemia and immune thrombocytopenic purpura: a Belgian retrospective multicentric study. J Inter Med.

[28] Michel M, Chanet V, Dechartres A (2009). The spectrum of Evans syndrome in adults: new insight into the disease based on the analysis of 68 cases. Blood.

[29] Berentsen S, Randen U, Vågan AM (2010). High response rate and durable remissions following fludarabine and rituximab combination therapy for chronic cold agglutinin disease. Blood.

[30] Peñalver FJ, Alvarez-Larrán A, Díez-Martin JL (2010). Rituximab is an effective and safe therapeutic alternative in adults with refractory and severe autoimmune hemolytic anemia. Ann Hematol.

[31] Rossignol J, Michallet AS, Oberic L (2011). Rituximab-cyclophosphamide-dexamethasone combination in the management of autoimmune cytopenias associated with chronic lymphocytic leukemia. Leukemia.

[32] Barcellini W, Zaja F, Zaninoni A (2012). Low-dose rituximab in adult patients with idiopathic autoimmune hemolytic anemia: clinical efficacy and biologic studies. Blood.

[33] Barcellini W, Zaja F, Zaninoni A (2013). Sustained response to low-dose rituximab in idiopathic autoimmune hemolytic anemia. Eur J Haematol.

[34] Birgens H, Frederiksen H, Hasselbalch HC (2013). A phase III randomized trial comparing glucocorticoid monotherapy versus glucocorticoid and rituximab in patients with autoimmune haemolytic anaemia. Br J Haematol.

[35] Maung SW, Leahy M, O’Leary HM (2013). A multi-centre retrospective study of rituximab use in the treatment of relapsed or resistant warm autoimmune haemolytic anaemia. Br J Haematol.

[36] Barcellini W, Fattizzo B, Zaninoni A (2014). Clinical heterogeneity and predictors of outcome in primary autoimmune hemolytic anemia: a GIMEMA study of 308 Patients. Blood.

[37] Roumier M, Loustau V, Guillaud C (2014). Characteristics and outcome of warm autoimmune hemolytic anemia in adults: new insights based on a single-center experience with 60 patients. Am J Hematol.

[38] Quinquenel A, Willekens C, Dupuis J (2015). Bendamustine and rituximab combination in the management of chronic lymphocytic leukemia-associated autoimmune hemolytic anemia: a multicentric retrospective study of the French CLL intergroup (GCFLLC/MW and GOELAMS). Am J Hematol.

[39] Fu R, Yan S, Wang X (2016). A monocentric retrospective study comparing pulse cyclophosphamide therapy versus low dose rituximab in the treatment of refractory autoimmune hemolytic anemia in adults. Int J Hematol.

[40] Laribi K, Bolle D, Ghnaya H (2016). Rituximab is an effective and safe treatment of relapse in elderly patients with resistant warm AIHA. Ann Hematol.

[41] Berentsen S, Randen U, Oksman M (2017). Bendamustine plus rituximab for chronic cold agglutinin disease: results of a Nordic prospective multicenter trial. Blood.

[42] Ducassou S, Leverger G, Fernandes H (2017). Benefits of rituximab as a second-line treatment for autoimmune haemolytic anaemia in children: a prospective French cohort study. Br J Haematol.

[43] Serris A, Amoura Z, Canouï-Poitrine F (2018). Efficacy and safety of rituximab for systemic lupus erythematosus-associated immune cytopenias: a multicenter retrospective cohort study of 71 adults. Am J Hematol.

[44] Fakhouri F, Vernant JP, Veyradier A (2005). Efficiency of curative and prophylactic treatment with rituximab in ADAMTS13-deficient thrombotic thrombocytopenic purpura: a study of 11 cases. Blood.

[45] Jasti S, Coyle T, Gentile T (2008). Rituximab as an adjunct to plasma exchange in TTP: a report of 12 cases and review of literature. J Clin Apheresis.

[46] Ling HT, Field JJ, Blinder MA (2009). Sustained response with rituximab in patients with thrombotic thrombocytopenic purpura: a report of 13 cases and review of the literature. Am J Hematol.

[47] Chemnitz JM, Uener J, Hallek M (2010). Long-term follow-up of idiopathic thrombotic thrombocytopenic purpura treated with rituximab. Ann Hematol.

[48] Rubia JDL, Moscardó F, Gómez MJ (2010). Efficacy and safety of rituximab in adult patients with idiopathic relapsing or refractory thrombotic thrombocytopenic purpura: results of a Spanish multicenter study. Transf Apheresis Sci.

[49] Froissart A, Buffet M, Veyradier A (2012). Efficacy and safety of first-line rituximab in severe, acquired thrombotic thrombocytopenic purpura with a suboptimal response to plasma exchange: experience of the French Thrombotic Microangiopathies Reference Center. Crit Care Med.

[50] Westwood JP, Webster H, McGuckin S (2013). Rituximab for thrombotic thrombocytopenic purpura: benefit of early administration during acute episodes and use of prophylaxis to prevent relapse. J Thromb Haemost.

[51] Hie M, Gay J, Galicier L (2014). Preemptive rituximab infusions after remission efficiently prevent relapses in acquired thrombotic thrombocytopenic purpura. Blood.

[52] Khandelwal P, Gupta A, Sinha A (2014). Effect of plasma exchange and immunosuppressive medications on antibody titers and outcome in anti-complement factor H antibody-associated hemolytic uremic syndrome. Pediat Nephrol.

[53] El Omri H, Taha RY, Gamil A (2015). Efficacy and safety of rituximab for refractory and relapsing thrombotic thrombocytopenic purpura: a cohort of 10 cases. Clin Med Insights Blood Disord.

[54] Benhamou Y, Paintaud G, Azoulay E (2016). Efficacy of a rituximab regimen based on B cell depletion in thrombotic thrombocytopenic purpura with suboptimal response to standard treatment: results of a phase II, multicenter noncomparative study. Am J Hematol.

[55] Chen H, Fu A, Wang J (2017). Rituximab as first-line treatment for acquired thrombotic thrombocytopenic purpura. J Int Med Res.

[56] Razavi-Shearer D, Gamkrelidze I, Nguyen MH (2018). Global prevalence, treatment, and prevention of hepatitis B virus infection in 2016: a modelling study. Lancet Gastroenterol Hepatol.

[57] Raufi AG, Scott S, Darwish O, Harley K, Kahlon K, Desai S, Lu Y, Tran M-H (2016). Atypical hemolytic uremic syndrome secondary to lupus nephritis, responsive to eculizumab. Hematol Rep.

[58] Dolin HH, Dziuba M, Pappada SM, Papadimos TJ (2019). Presumed antiphospholipid syndrome and thrombotic thrombocytopenic purpura: an infrequent association. Clin Case Rep.

[59] Biljana M, Jelena M, Branislav J (1999). Bias in meta-analysis and funnel plot asymmetry. Stud Health Technol Inform.

